# Sex-Related Differences in the Maximal Lactate Steady State

**DOI:** 10.3390/sports6040154

**Published:** 2018-11-27

**Authors:** Paul S. Hafen, Pat R. Vehrs

**Affiliations:** Department of Exercise Sciences, 106 SFH; Brigham Young University; Provo, UT 84602, USA; basicrunner2002@yahoo.com

**Keywords:** competitive, running, runners, female, respiratory exchange ratio (RER), maximal lactate steady-state (MLSS)

## Abstract

The maximal lactate steady state (MLSS) is one of the factors that differentiates performance in aerobic events. The purpose of this study was to investigate the sex differences in oxygen consumption (VO_2_), heart rate (HR), and the respiratory exchange ratio (RER) at the MLSS in well-trained distance runners. Twenty-two (12 female, 10 male) well-trained distance runners (23 ± 5.0 years) performed multiple 30-min steady-state runs to determine their MLSS, during which blood lactate and respiratory gas exchange measures were taken. To interpret the MLSS intensity as a training tool, runners completed a time-to-exhaustion (TTE) run at their MLSS. The relative intensity at which the MLSS occurred was identical between males and females according to both oxygen consumption (83 ± 5 %O_2_max) and heart rate (89 ± 7 %HRmax). However, female runners displayed a significantly lower RER at MLSS compared to male runners (*p* < 0.0001; 0.84 ± 0.02 vs. 0.88 ± 0.04, respectively). There was not a significant difference in TTE at MLSS between males (79 ± 17 min) and females (80 ± 25 min). Due to the observed difference in the RER at the MLSS, it is suggested that RER derived estimates of MLSS be sex-specific. While the RER data suggest that the MLSS represents different metabolic intensities for males and females, the relative training load of MLSS appears to be similar in males and female runners.

## 1. Introduction

Performance in aerobic events is largely dependent on maximal sustainable power or velocity. The oxygen consumption (VO_2_) at this velocity is referred to as the performance VO_2_ [1], and is primarily determined by the maximal lactate steady state (MLSS) or lactate threshold [2]. The velocity at the performance VO_2_ is the best physiological predictor of distance running performance [2]. Unfortunately, the determination of the MLSS is laborious, as the associated blood lactate analysis requires the collection of multiple blood samples multiple 30-min exercise tests. Thus, the development of less demanding methods to estimate the MLSS would be beneficial.

The respiratory exchange ratio (RER) is typically used to estimate caloric expenditure and oxidative substrate utilization [3]. The RER can also be used to identify differences in training status and muscular efficiency [4,5]. Key factors that influence the RER during exercise include intensity of exercise, muscle fiber composition, and the timing and composition of pre-exercise food consumption [6]. Previous reports [7,8] indicate that during submaximal-intensity aerobic exercise, females demonstrate lower RER values and higher fat utilization rates than their male counterparts.

Previous studies have observed RER values near 1.0 at intensities corresponding to the MLSS [9,10]. Thus, it has been suggested that an intensity of exercise corresponding to an RER = 1.0 may represent a practical, non-invasive approximation of the intensity of exercise corresponding to the MLSS [11,12]. Despite evidence that submaximal steady-state RER is influenced in part by training volume [6] and sex [7], most of the research investigating the relationship between the RER and the MLSS has been done exclusively in either untrained [13], recreationally active [11,14,15], and/or well-trained [9,12,16,17,18] males. One study [19] that investigated sex-related differences in the relationship between the RER and the MLSS used a heterogeneous group that participated in a variety of endurance, strength training, and other physical activities, without specific reports on the training status or aerobic capacity (VO_2_max) of the participants. Therefore, the purpose of this study was to investigate whether common physiological variables (VO_2_, heart rate (HR), RER) at the MLSS might be influenced by sex in a more homogeneous group of well-trained distance runners. We hypothesized that females would exhibit a lower RER at the MLSS, and that this lower RER would improve MLSS performance by prolonging the time-to-exhaustion (TTE) while running at the velocity associated with the MLSS (vMLSS).

## 2. Methods

### 2.1. Subjects

Twelve female (21.1 ± 3.9 years, 167.9 ± 7.0 cm, 58.5 ± 5.2 kg) and 10 male (27.2 ± 3.8 years, 179.6 ± 8.1 cm, 72.6 ± 5.2 kg) runners voluntarily participated in this study. To establish training status, all participants had [1] a 5000 m personal best time that was within 30% of the current world record time (i.e., 18.00 min for males and 20.25 min for females), and [2] been running at least 4–8 miles per training session for 3 or more days/week over the preceding 6 months. All participants provided written, informed consent after all procedures and potential risks and benefits were explained. This study was approved by the Institutional Review Board for the use of human subjects.

Female runners were tested within the first 14 days of their menstrual cycle, defined as the follicular phase of menses. This was done to minimize the potential confounding effects of intra-individual hormone variability through the menstrual cycle, characterized by elevated estradiol levels during the later phases of menses. Such increases in estradiol have been directly linked to reduced blood lactate concentrations and increased fat oxidation rates during submaximal exercise [20,21,22].

### 2.2. Experimental Design

This study consisted of 3–6 exercise-testing sessions over the course of 10–14 days. During the first session, participants completed a maximal treadmill graded exercise test (GXT) to determine their VO_2_max. During subsequent exercise testing sessions, participants completed 30-min exercise tests at constant treadmill speeds to determine their individual MLSS. All runners completed each of the running trials following an overnight fast. Once the MLSS was identified, a time to exhaustion (TTE) test was completed at the vMLSS.

### 2.3. Methodology

#### 2.3.1. Maximal Exercise Testing

All participants completed a treadmill (Trackmaster TMX425L, Full Vision Inc., Newton, KS, USA) GXT beginning at a speed of 9.7 km∙hr^−1^ (6 mph) with a 1.5% grade. The grade remained constant throughout the test and the treadmill speed was increased by 1 km∙hr^−1^ (0.6 mph) every 3 min until subjects fatigued and voluntarily terminated the exercise test despite verbal encouragement. Respiratory gas exchange variables were measured continuously (TrueOne 2400, ParvoMedics, Sandy, UT, USA). Heart rate (HR) was measured using a chest-strap HR monitor (Polar Electro OY, Hong Kong). Ratings of perceived exertion (RPE) were monitored at the end of each 3 min stage using the Borg 15-point scale [23].

Each runner’s effort during the GXT was considered maximal if there was a plateau in oxygen consumption (VO_2_) despite an increase in workload at the final stage. A plateau in VO_2_ was defined as an increase in VO_2_ of less than one-half of the increase expected from the increase in speed. In the absence of a plateau, two of the following criteria were considered requisite for the determination of aerobic capacity: (1) a maximal RER ≥ 1.10, (2) a maximal HR greater than 90% of age-predicted maximal HR (208 − (0.7 × age)), and/or (3) a final RPE ≥ 18.

#### 2.3.2. MLSS Testing

During the MLSS running trials, respiratory gas exchange variables and HR were continuously monitored as described for the GXT. The MLSS was defined as the highest steady-state blood lactate concentration (cMLSS) and corresponding vMLSS that could be achieved during a 30 min running trial at a constant speed. Blood lactate concentrations were considered steady-state if blood lactate increased by less than 1 mmol between the 10th and 30th min of the 30 min running trial. The cMLSS and vMLSS were identified from multiple 30 min submaximal running trials completed on separate days. The running trials began three to four days following the maximal GXT and each submaximal running trial was separated by 48 h. For each MLSS trial, an indwelling venous catheter was inserted into an antecubital vein in order to obtain small (≈2 mL) blood samples at 5 min intervals without interrupting the 30 min running trials.

Plasma lactate concentrations for each of the running trials were obtained using a calibrated clinical grade lactate analyzer (YSI 2300 STAT Plus™, YSI Life Sciences, Yellow Springs, OH, USA). Briefly, approximately 135 µL of blood was separated from each sample for centrifugation in order to obtain triplicate measures of hematocrit. An additional 60 µL was separated from each blood sample for triplicate measures of hemoglobin content according to the standard clinical technique using a cyanomethemoglobin reagent via a photometric multilable counter (VICTOR^3^ 1420 Multilabel Counter, PerkinElmer, Waltham, MA, USA). The remainder of each sample was centrifuged for plasma lactate analysis in the YSI 2300 STAT Plus™. Each plasma lactate sample was adjusted to account for any shifts in blood volume that might occur due to the duration of each running trial [24].

The initial MLSS running speed was determined following the GXT, beginning at a velocity associated with 80% of the participant’s VO_2_max. Following the initial running trial, running speed was adjusted appropriately for subsequent trials. If, during the preceding running trial, plasma lactate concentration increased by more than 1 mmol between the 10th and 30th min, the running speed during the subsequent trial was decreased by 0.6 km∙hr^−1^ (0.4 mph). Conversely, if the lactate concentration increased by less than 1 mmol between the 10th and 30th minute, the speed of the subsequent trial was increased by 0.6 km∙hr^−1^ (0.4 mph). This protocol allowed for the determination of cMLSS and vMLSS in as few as two, but no more than four, 30 min running trials for all runners.

To test the notion that an intensity of exercise eliciting an RER = 1.0 corresponded to the MLSS, and to determine the duration that this intensity could be maintained, a subset of 8 runners began MLSS testing at an intensity of exercise that elicited an RER as close as possible to 1.0. The speed of the treadmill was adjusted during the first several minutes of the exercise test to allow the participant to achieve an RER near 1.0. The exercise test progressed without any further changes in treadmill speed and the participant was then encouraged to run as long as possible.

#### 2.3.3. TTE Testing

Approximately 48 h following the final MLSS running trial, each runner completed a TTE running trial at their personal vMLSS. Fluid ingestion was not permitted during the TTE running trial. Each participant received verbal encouragement from the researchers and were instructed to run at the assigned speed for as long as they were able. The TTE trial ended when participants felt unable to continue running at their vMLSS.

### 2.4. Statistical Analysis

A two-way mixed analysis of variance (ANOVA) was used to compare the physiological data from the MLSS trials in order to properly account for variability between groups (male vs. female) over time (5 min intervals). The mixed ANOVA was the appropriate test for our analyses as each subject had multiple observations. Group means were compared using independent t-tests. All analyses were completed using SAS JMP^®^ Pro 12.0.1 statistical software. The alpha level for statistical analysis was set at 0.05.

## 3. Results

Table 1 includes the self-reported training volume, 5000 m personal best times, and the results from the GXT. Five (50%) males and 8 (67%) females demonstrated a VO_2_ plateau on the GXT. All other runners met other criteria for maximal effort. Compared to females, the VO_2_max in males was 35% higher (*p* < 0.05) when expressed in absolute (L·min^−1^) terms and 7% higher when expressed relative to (mL·kg^−1^·min^−1^) body mass. There were no differences (*p* > 0.05) in maximal HR or RER between the male and female runners.

**Figure 1:**
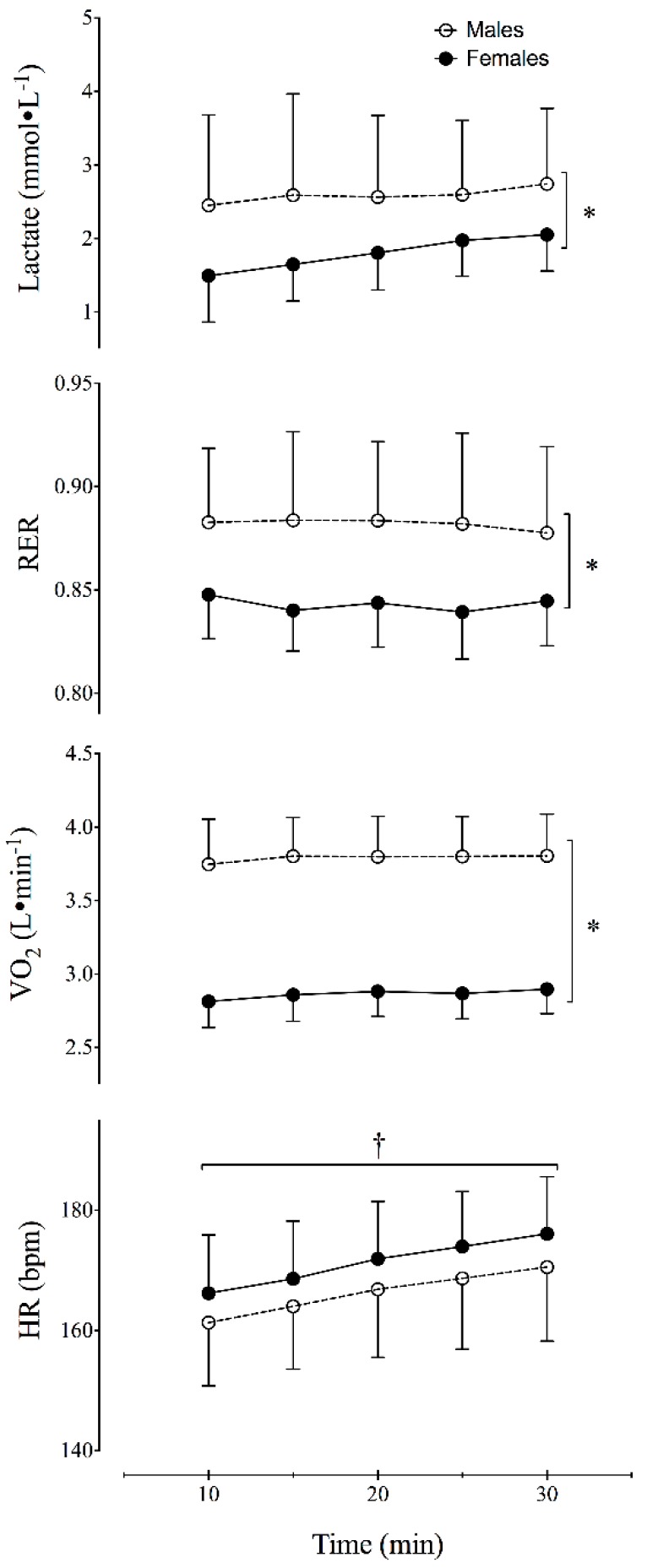
Time course of physiological variables during the MLSS running trial. Values reported are means ± SD. * Significant difference (*p* < 0.05) between males and females. ^†^ Significant change (*p* < 0.05) over time.

Table 2 summarizes the results of the MLSS running trial. The cMLSS was significantly lower (*p* = 0.0402) in the female runners (1.79 ± 0.49 mmol∙L^−1^) compared to the male runners (2.59 ± 1.14 mmol∙L^−1^; Table 2, Figure 1). The RER at the MLSS in female runners (0.84 ± 0.02) was also significantly lower (*p* < 0.0001) than the RER observed in the male runners (0.88 ± 0.04) (Table 2, Figure 1). At the MLSS, RER was significantly lower than 1.0 (*p* < 0.0001) for both male and female runners. Additionally, there was not a significant effect of time on RER (*p* = 0.6631) during the MLSS trial.

The MLSS occurred at similar relative intensities in males and females (83 ± 4.6 and 83 ± 6.7%VO_2_max, respectively). Additionally, both groups demonstrated steady-state VO_2_ kinetics during the MLSS trials (Figure 1) as the VO_2_ did not change significantly (*p* = 0.3739) between the 10th and 30th minutes of the vMLSS trial. The relative HR (%HRmax) at the MLSS was also similar between males and females (89.5 ± 8.8 and 88.6 ± 6.8%HRmax, respectively). In both groups, HR increased significantly (*p* = 0.0007) over time during the vMLSS trial (Figure 1). This rate of increase was approximately 0.48 bpm (95% CI: 0.21 to 0.76 bpm).

Of the subset of 8 participants who ran at a treadmill speed representing the vRER = 1.0, 7 achieved RER values between 0.97 and 0.99 and ran for 9.4–15.25 min. One participant achieved an RER = 1.05 and was only able to run for 6 min before voluntarily terminating the test due to fatigue. Finally, there was not a significant difference (*p* = 0.4580) in TTE between the male (79 ± 17 min) and female runners (80 ± 25 min) at the vMLSS (Table 2).

## 4. Discussion

Previous research suggested that the vMLSS may be similar to the speed at which RER = 1.0 during a maximal GXT [11,12,25,26]. The results of this study (Table 2; Figure 1) indicate that the RER at the vMLSS is considerably less than 1.0 in well-trained runners. Additionally, the RER at the MLSS was significantly lower in female runners (0.84 ± 0.02) compared to male runners (0.88 ± 0.04). The results of this study suggest that the use of an RER value to estimate the vMLSS varies depending on the sex and training status of the athlete.

The lower RER values at the vMLSS in the female runners in this study are supported by the tendency of females to exhibit lower RER values and greater rates of fat oxidation at a given relative sub-maximal intensity of exercise [8]. Additional experimental research involving sex-hormone supplementation in men has recently begun to unravel the particular mechanisms of observed sex differences in substrate utilization during exercise. For example, when supplementing men with estrogen, lower RER values have been observed both at rest and during exercise [27]. Furthermore, this type of supplementation seems to be regulating the expression of genes involved in the oxidation of fat, such as peroxisome proliferator-activated receptor γ coactivator-1α (PGC-1α) and microRNA-29b [28]. Therefore, it appears as though the lower RER, and increased fat oxidation, observed during submaximal exercise in females may be heavily influenced by sex hormones.

The lower RER observed at the vMLSS in the well-trained female runners in our study was also accompanied by a lower cMLSS (Table 2, Figure 1). The findings of this study are contrary to a recent report [19] that the RER and blood lactate concentrations did not differ between men and women while cycling at the MLSS intensity. The reported blood lactate levels for their male and female participants (4.80 ± 1.50 mmol∙L^−1^ and 5.22 ± 1.52 mmol∙L^−1^, respectively) are higher than the concentrations observed in this study (Table 2). Participants in the previous study [19] represented a heterogeneous group of males and females who reported a weekly combined training volume of 0-24.5 h/week in a variety of endurance activities (running, cycling, and swimming), resistance exercise, game sports (e.g., tennis, badminton, soccer, volleyball) and various other physical activities (e.g., dance, canoeing). Therefore, we believe that the heterogeneous nature of the participants training background prevented the researchers from identifying sex-related differences in the RER and cMLSS.

The mean blood lactate concentrations at the vMLSS for males have ranged between 3 and 6 mmol∙L^−1^ depending on the mode of exercise [9,11,12,14,29,30]. Although Beneke et al. [14] reported an average cMLSS of 4.9 ± 1.5 mmol∙L^−1^ during cycling in 33 male participants, the cMLSS ranged from 1.9 to 7.5 mmol∙L^−1^. This wide range of cMLSS was likely due to the fact that 10 of the participants were trained cyclists while the remaining 23 participants were not trained. Our attempt to preserve homogeneity within groups likely decreased the variability of both the RER and cMLSS measures, minimizing the potential confounding effects of training status and endurance capacity.

Such discrepancies in the results of this study compared to previous studies may also be attributed to the methods used to measure blood lactate. In this study, plasma blood lactate was measured in triplicate from a blood sample drawn from an intravenous catheter. Other studies have measured blood lactate from a single 20–25 µL whole blood capillary blood sample taken from the ear lobe either during a 15–30 s rest period between each 5 min interval of exercise [9,12], or while the participant was exercising [11,14,17,29]. Such measurement techniques may contribute to differences between studies as we used centrifuged blood taken from the arm via an intravenous catheter and corrected for potential fluctuations in the hemodynamics associated with exercise [24].

Previous research tested the hypothesis that the intensity of exercise associated with an RER = 1.0 was an accurate estimate of the intensity of exercise associated with the MLSS during leg ergometry [11] and running [12]. The cycling workload [11] and running speed [12] associated with an RER = 1.0 during an incremental maximal exercise test of 1-min stages was used to estimate the intensity of exercise corresponding to the MLSS, despite, as the authors admit [11], insufficient time during each stage to assure steady-state gas exchange data. Laplaud et al. [11] reported that the RER remained near 1.0 during the MLSS exercise trial and that the MLSS workload corresponded to a similar workload during the maximal exercise test when RER = 1.0. For the participants in our study, running at an RER = 1.0 would represent an intensity of exercise at or above their VO_2_max in 9 of the 22 participants. Furthermore, subjects in this study were only able to run at the vRER = 1.0 (RER = 0.97 − 1.05) for an average of 10.35 min. Thus, our data are contrary to reports that intensities of exercise eliciting an RER ≈ 1.0 can be maintained for prolonged periods of time [11,12,26,31]. The RER values recorded in the male runners in this study at the vMLSS (RER = 0.88) are more in line with previously reported values (RER ≈ 0.93) at the vMLSS in well-trained male runners [17] and cyclists [32].

It is important to note that much of the previous research [11,12,17] on RER at the MLSS did not describe any pre-exercise dietary controls aside from advising participants to maintain their regular diet the day prior to testing and to report to the lab at the same time of day for each test. As exercising in the fed state results in higher rates of carbohydrate oxidation, and therefore a higher RER during exercise [4], it is reasonable to suggest that the lower RER values observed at the MLSS in the runners in this study may be due to performing all exercise tests in a fasted state.

Although the exercise test to determine the cMLSS and vMLSS is 30 min in duration, it is presumed that the vMLSS can be maintained for extended durations [16] and is predictive of performance in all endurance-type sports [26]. Our results were not in support of our second hypothesis that a lower RER in females would be accompanied by superior endurance time at the vMLSS. The TTE at the vMLSS reported in this study (Table 2) ranged from 40 min to 97 min in males and from 40 min to 120 min in females. These times are considerably greater than the 35 min previously reported in moderately trained participants during cycling and running [33]. In support of our observations, more recent studies have reported a TTE at MLSS ranging from approximately 54 min [18] to 70 min [13] during cycling.

Complicating our current understanding of the particular mechanisms of fatigue at the MLSS is the concept that fatigue during prolonged exercise can be attributed to an interplay of various factors including: energy reserve status [34], hydration status [35], body temperature [36], the accumulation of lactate and H^+^ [37], and central motor command [38]. Not surprisingly, research by Baron et al. [39] was unable to associate fatigue at the MLSS with evidence of failure in any single physiological system. Given the variability in the observed TTE in this study, and between other studies, we suspect the mechanism of fatigue to be different between individuals. Also, as our TTE is among the highest reported, we suspect that most individuals may experience long-term training adaptations to promote a higher TTE at the vMLSS.

Future research into individual mechanisms of fatigue at the MLSS may be beneficial to both coaches and athletes in order to effectively utilize and manipulate this intensity as part of a specific exercise regimen. Perhaps future research involving the measure of MLSS and TTE over longer periods of training may shed light on these discrepancies and aid in the understanding of the MLSS as an exercise intensity, as it relates to fatigue and performance.

## 5. Conclusions

Our findings suggest that the metabolic intensity of MLSS is sex-specific, with female runners exhibiting lower RER values, higher fat oxidation rates, and lower plasma lactate concentrations, despite working at a similar relative intensity of exercise (%VO_2_max) as their male counterparts. We suggest that estimates of the vMLSS from gas exchange variables should also be sex-specific. However, these differences do not seem to influence the interpretation of the MLSS as a general training intensity in well-trained runners as %VO_2_max, %HRmax, and TTE at vMLSS were identical between male and female runners. Thus, the application of MLSS as a training tool may revolve around similar relative intensities and durations independent of sex.

## Figures and Tables

**Table 1:** Maximal Exercise Test Results.

	Males (n = 10)	Females (n = 12)
5000 m Best Time (min)	16.8 ± 0.8	17.7 ± 1.1 *
Training Volume (km·wk^−1^)	67.9 ± 23.0	85.8 ± 28.0
VO_2_max (L·min^−1^)	4.5 ± 0.3	3.4 ± 0.2 *
VO_2_max (mL·kg^−1^ ·min^−1^)	62.9 ± 4.5	59.0 ± 3.2 *
HRmax (bpm)	186.4 ± 10.5	193.8 ± 9.9
RERmax	1.01 ± 0.03	1.00 ± 0.03

Values reported are means ± SD. * Significant difference (*p* < 0.05) between males and females.

**Table 2:** Maximal Lactate Test Results.

	Males (n = 10)	Females (n = 12)
VO_2 (_mL·kg^−1^·min^−1^)	52.4 ± 3.8	49.1 ± 2.9
VO_2_ (%VO_2_max)	83.3 ± 4.6	83.0 ± 6.7
cMLSS (mmol·L^−1^)	2.59 ± 1.14	1.79 ± 0.49 *
HR (bpm)	166.3 ± 11.2	171.3 ± 9.4
HR (%HRmax)	89.5 ± 8.8	88.6 ± 6.8
RER	0.88 ± 0.04	0.84 ± 0.02 *
TTE (min)	79.0 ± 17.0	80.0 ± 25.1

Values reported are means ± SD at the maximal lactate. cMLSS = plasma lactate concentration at MLSS. TTE = time to exhaustion at the MLSS. * Significant difference (*p* < 0.05) between males and females.
